# Social Behaviour in Zoo Bachelor Groups: A Case Study of Related South American Fur Seals

**DOI:** 10.3390/ani11092682

**Published:** 2021-09-13

**Authors:** Christa Emmett, Mathilda Digby, Jemma Pope, Ellen Williams

**Affiliations:** 1 Department of Field Conservation & Science, Bristol Zoological Society, Bristol BS8 3HA, UK; christa.emmett@googlemail.com or; 2 Department of Applied Sciences, University of the West of England, Bristol BS16 1QY, UK or Mathilda2.Digby@live.uwe.ac.uk (M.D.); jemmapope96@gmail.com (J.P.)

**Keywords:** South American fur seals, *Arctocephalus australis*, social behaviour, bachelor groups, social needs, evidence-based management

## Abstract

**Simple Summary:**

Appropriate management of social groups is one of the greatest challenges that face zoos and aquaria worldwide. All-male social groups provide an opportunity for facilities to house surplus males in groups which optimise their welfare whilst they are being retained for future use in breeding programmes. Here, we investigated social behaviour in a relatively poorly studied species, the South American fur seal (SAFS). Four individuals housed in a related group were studied over a 6-month period. The results showed that their social relationships changed over time, although the individuals always engaged in more positive than negative interactions. We recommend establishing baseline social behaviour profiles of individuals to enable long-term monitoring of SAFS social groups, as has been recommended in other species. This will enable enhanced understanding of South American fur seals and will contribute to the development of evidence-based social management guidelines for this species.

**Abstract:**

Appropriate management of social groups is one of the greatest challenges that face zoos and aquaria worldwide. To facilitate breeding programmes, particularly in polygynous species, there is a need to house surplus males in bachelor groups, yet for pinnipeds, the social impact of this management strategy is unknown. The aim of this research was to enhance understanding of sociality in South American fur seals (SAFSs), with a particular focus on social dynamics in a related bachelor group, and consider implications for evidence-based management of this species in zoos. The subjects were four related male seals housed at Bristol Zoo Gardens. Social interaction and nearest neighbour data were collected between February and July 2019. Individuals engaged in both positive and negative social interactions. Positive interactions were more frequent than negative interactions, and no excessive negative interactions were observed. Temporal dynamics were observed in social relationships, and negative interactions did not increase with the onset of the breeding season. Reciprocity in dyadic relationships was variable across the study months, and nearest neighbours were not necessarily reflective of social partners. This research highlights the importance of longitudinal monitoring of social relationships and establishment of baseline social behaviour profiles to support evidence-based species management. We advocate that this research is extended, to further develop our understanding of SAFS social needs within zoo environments, to understand the differences between single-sex and mixed-sex groups and to identify the degree to which the extensive research conducted in other polygynous species (e.g., gorillas) is applicable in the social management of South American fur seals moving forwards.

## 1. Introduction

Pinnipeds exhibit intricate and complex social systems [1]. Generally, non-breeding bulls and sub-adult males form bachelor groups in the vicinity of breeding colonies [2,3]. The South American fur seal (*Arctocephalus australis*) is no exception. It is an eared seal found along the Atlantic and Pacific coasts of South America [4]. They reside in groups of 14 to 12,955 individuals (average 1883), with variation seen throughout the year [5]. They are poorly studied in comparison to other pinniped species [6], and there is a paucity of literature on their social dynamics, however, it is known they have a predominantly polygynous social structure and breed in the spring/summer (October to December) [7]. Males are either territorial or satellite. Satellite males remain near breeding groups but interactions between individuals are lower than between dominant males [8]. Within zoos in the northern hemisphere, the otariid breeding season is usually spring/summer (May to August), but there may be an additional breeding season in December [9]. Sexual maturity is generally between the ages of four and seven, although otariids have been recorded as able to sire/carry offspring from the age of two years within zoos [9].

Traditionally, there were difficulties in catering for polygynous species in zoos [10]. Non-breeding males usually could not be maintained within breeding groups due to competition for mates [11]. Bachelor groups naturally occur in the wild in a number of polygynous species [3]. However, these groups may be relatively unstable, with individuals migrating once they reach sexual maturity [12]. Formation of bachelor groups in zoos provides an opportunity to manage these surplus males (both adults and sub-adults) [13]. Although a relatively recent development in zoos, bachelor groups have been identified as a successful solution to housing excess males [11,14], despite concerns of potential for aggression and wounding within groups [12].

Appropriate social groups are a form of social enrichment and support animals to develop species-specific behaviour [15]. Social interactions are recognised as a good source of enrichment for zoo-housed pinnipeds [9]. However, appropriate management of social groups is one of the greatest challenges that face zoos and aquaria today [16]. Group social stability is considered crucial to successful social management, however, sometimes zoo management practices (e.g., introduction of new social members, separation for individual training regimes) or other natural changes to social groups (e.g., the death of a social member) can disturb social groups [12]. A comprehensive understanding of species-specific social dynamics can help to mitigate management-related social disturbance [17]. Understanding natural fluctuations in social behaviour and temporal dynamics in relationships has been identified as a useful tool in evidence-based social management approaches [18]. Effective population management of social, in particular polygynous, species requires successful formation and maintenance of compatible bachelor groups. A plethora of work has been undertaken in primates to establish baseline knowledge in relation to the management of bachelor groups and to consider their management differences in relation to mixed-sex groups [12]. Expanding this knowledge into other social species is important for improved animal welfare across taxa.

Approximately 71% of marine mammals housed in zoos are pinnipeds [19], yet there is a paucity of research on pinniped social groupings, especially in terms of bachelor groups. The European Association of Zoos and Aquaria (EAZA) guidelines on the management of captive pinnipeds suggest that groups should comprise a variety of age groups, as an unbalanced age structure can create disruptive hierarchies [9]. However, this recommendation may not be practical or relevant for single sex, non-breeding groups. Within ZIMS-registered collections, SAFSs (*n* = 58) are held at 20 facilities worldwide. Bristol Zoo is one of only three holders of bachelor groups of SAFSs. There are 4 female-only groups and 13 mixed-sex groups. Groups range in size from 1 to 7 individuals (mean of 2.9) [20]. The expression of natural behaviours (e.g., play and socialising) may improve in multi-male groups, if enough room is provided for choice of when to interact with or avoid conspecifics [9]. The development of evidence-based social group recommendations requires a baseline knowledge of animal social needs. Such recommendations should depend on whether individuals are housed in a single- or mixed-sex exhibit, and will likely be affected by the individuals in the group.

Pinniped social group composition has been highlighted as a required area for research, in order to advance species knowledge and improve animal management [9]. To the authors’ knowledge, at the time of writing there was no published research on social behaviour in bachelor groups of SAFSs in zoos. Capturing data on temporal dynamics is important in understanding animal social networks [21]. The aim of this research was to enhance understanding of sociality in SAFSs, with a particular focus on social dynamics in a related bachelor group, and to consider implications for evidence-based management of this species in zoos. Furthermore, it was to understand whether bachelor groups of SAFSs show temporal dynamics in their relationships which align with the breeding season. This knowledge will enable enhanced understanding of SAFS social relationships in zoos and proactive management approaches which ensure positive welfare.

## 2. Materials and Methods

### 2.1. Study Subjects and Enclosure

**Figure 1:**
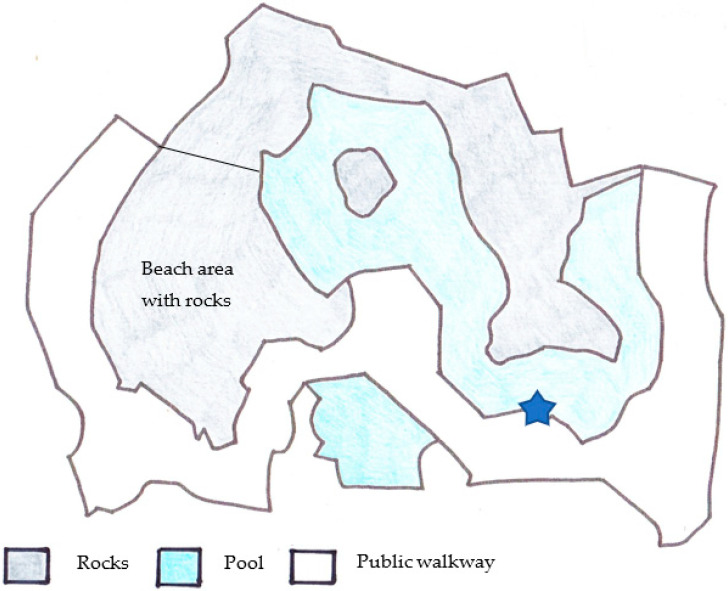
A schematic drawing of the South American fur seals enclosure at Bristol Zoo Gardens [23]. Blue star indicates where observations were made from.

The study subjects were four related male SAFSs (Table 1), all of whom were captive born. Individuals were housed in the Seal and Penguin Coasts exhibit at Bristol Zoo Gardens [22], in an enclosure (160 m^2^) that consisted of two pools (total surface area approximately 80 m^2^, volume approximately 450 m^3^ and 2.4–3 m deep), external rocks (approximately 80 m^2^) and an indoor shelter. There were no female SAFSs housed at the zoo, nor were there any other pinniped species. All observations were made from a viewing deck above the surface of the water (Figure 1). Random feeds occurred twice per day, as well as a daily public talk and feed occurring at 3:30 p.m.

### 2.2. Data Collection

Preliminary observations to identify individual seals and refine the ethogram and data collection methods were undertaken ad libitum during January 2019. Seals were identified using visually discernible differences. Data were collected from February to July 2019, on two randomly selected days per week (Monday to Friday) between 09:00 and 16:00. Where possible, two observation sessions (1 × AM and 1 × PM) were undertaken per day. Each observation session lasted 1 h. A total of 98 observation periods were undertaken over 50 days.

Due to the small number of seals in the group (*n* = 4), data were collected on the entire group simultaneously. During each 1 h observation period, social interactions were captured using a behaviour sampling method with continuous (all-occurrence) recording [24]. When social interactions were recorded, the following data was collected: individuals involved, direction of interaction (e.g., unidirectional or bidirectional) and nature of the interaction (positive or negative) (Table 2). Scan sampling (5 min inter-scan interval) with an instantaneous recording method was used to gather data on proximity to other seals (identity of closest seal or seals) [24]. If seals were not within two body lengths of another seal at the time of observation they were classed as being ‘alone’.

### 2.3. Data Analysis

Data analysis was split into two areas: (i) frequency of social interactions given by individual seals and within-seal dyads; (ii) group social matrices (group size *n* = 4 seals). Data were split into months of data collection (February–July), to investigate the stability of group dynamics over time.

#### 2.3.1. Frequency of Social Interactions

To understand the role of individuals within the social network, centrality measures were calculated using NetDraw Version 2.160 [26]. Degree centrality has been identified as a useful approximation of individual centrality, which shows high correlations with other centrality measures [27]. Due to the small network size, this was considered an appropriate measure of centrality for this simple network. Betweenness centrality has been used to identify how important individuals are in terms of network cohesion [28]. This was additionally calculated to determine whether any individuals were considered ‘key’ in the social group, based on social interactions.

Statistical analysis was undertaken in R Studio (Version 4.0.3) [29], using packages ‘lme4′ [30], ‘emmeans’ [31] and ‘MASS’ [32]. Graphs were produced using ‘ggplot2′ [33]. All model results are reported as model estimate (β1) ± SE. Significance values were set at *p* < 0.05 for all analyses.

A wilcoxon test for paired samples was used to assess the relationship between the frequency of positive and negative social interactions at each observation period. Negative binomial general linear models (GLMs) were used to investigate differences in social interactions given by individual seals. Frequency of positive and negative social interactions was fitted as response variables and seal was fitted as a fixed effect. General linear mixed models (GLMMs), with Tukey-corrected post-hoc tests where appropriate, were used to investigate whether the frequency of social interactions given by individual seals and within seal dyads changed over time (study months February to July). Frequency of positive and negative interactions given by individual seals and within seal dyads were fitted as response variables in two separate models. Observation month was fitted as a fixed effect. To control for repeated observations, seal was included as a random effect in each model.

#### 2.3.2. Assessment of Group-Level Interactions Using Social Matrices

Changes in seal relationships within the social group (using interaction frequencies and nearest neighbour preferences) over time and reciprocity in dyads were assessed using Mantel tests using package ‘vegan’ [34]. A total of 999 permutations were used per test, with the Pearson product moment correlation coefficient as the test statistic. Significance levels were set at 0.05.

Social interaction matrices were created using frequency of interaction data for positive and negative interactions, and nearest neighbour data. Matrices of total frequency of interactions and proximity to others were created for each month. All months were compared against all months (February to July) to establish whether social group structure differed throughout the study period. Each month was then compared with the subsequent month (February–March; March–April; April–May; May–June; June–July) to examine changes in group structure over a longitudinal period. Correlation between the matrices indicated consistency in social networks.

Mantel tests were then used to assess whether nearest neighbour data was reflective of social interaction partners each month. No correlation between the two matrices indicated that the two networks (social interaction and nearest neighbour) were not representative of one another.

Tests of reciprocity were undertaken to determine whether dyadic social interactions were reciprocal (i.e., to determine whether the rate of interaction S1 directed towards S2 was correlated with the rate of interaction that S2 directed to S1). No correlation between the matrix and its transpose indicated unidirectional interactions.

## 3. Results

### 3.1. Frequency of Social Interactions

#### 3.1.1. Interactions Given by Individual Seals

**Figure 2:**
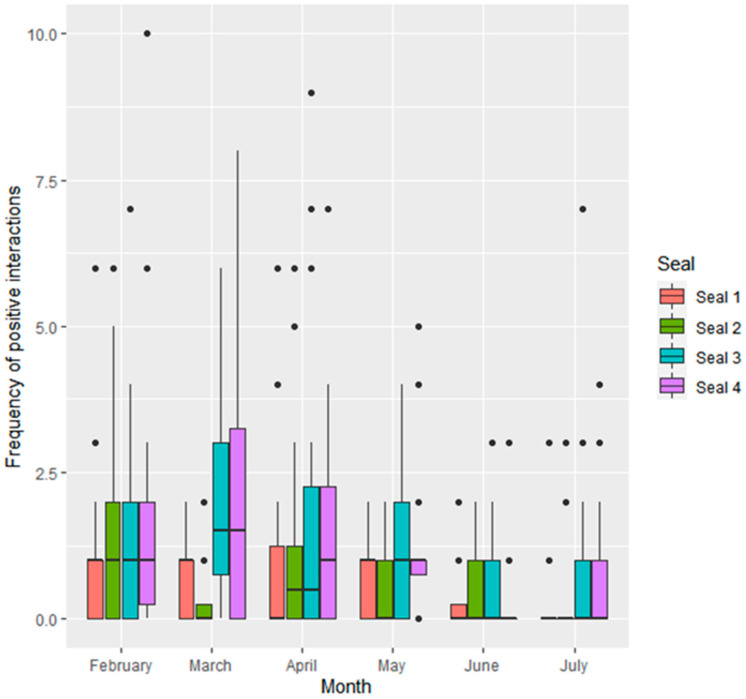
Frequency of positive social interactions given by each seal (*n* = 4) per observation period.

**Figure 3:**
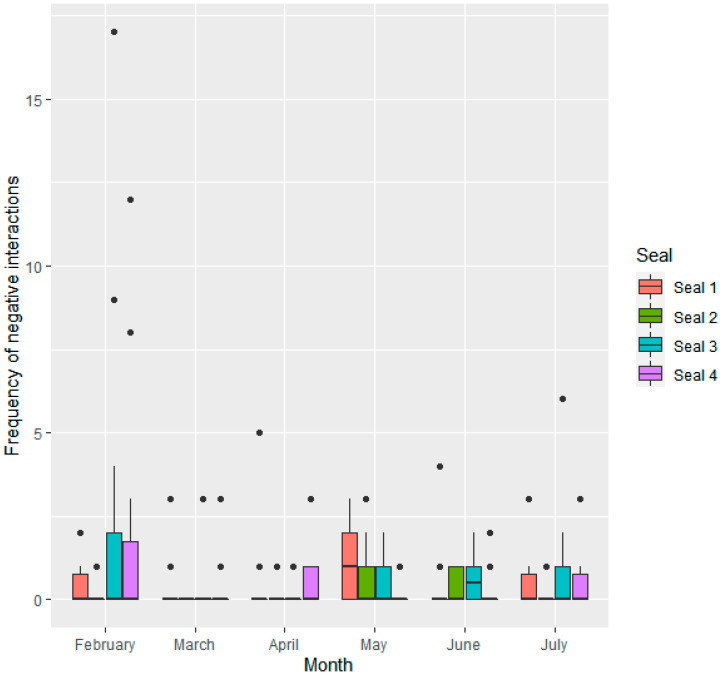
Frequency of negative social interactions given by each seal (*n* = 4) per observation period.

All seals engaged in both positive and negative social interactions with all other seals. Seals had equal centrality within both the positive and negative social network (degree centrality score = 3, betweenness centrality score = 0). Within each observation period, seals performed significantly more positive (mean observations ± SD per 1 h observation period; 1.06 ± 1.6) than negative (0.49 ± 1.4) social interactions (W = 18,496, *p* < 0.001). Across all study months, there were significant differences between seals in frequency of positive (Figure 2) and negative (Figure 3) social interactions.

Seal 3 engaged in more positive social interactions than seal 1 (0.67 ± 0.22, Z = 3.121, *p* < 0.01) and seal 2 (0.55 ± 0.21, Z = 2.605, *p* < 0.05). Seal 4 also engaged in more positive social interactions than seal 1 (0.68 ± 0.22, Z = 3.156, *p* < 0.01) and seal 2 (0.56 ± 0.21, Z = 2.641, *p* < 0.05). There were no significant differences between seal 1 and seal 2 (*p* > 0.05), or seal 3 and seal 4 (*p* > 0.05). Seal 3 and seal 4 engaged in more negative social interactions per observation period than seal 2 (respectively, 1.32 ± 0.36, Z = 3.651, *p* < 0.01; 0.99 ± 0.37, Z = 2.691, *p* < 0.05).

Frequency of interactions given by seals differed across the months. Frequency of positive social interactions per sampling period was significantly lower in June than in February (−1.06 ± 0.27, Z = −3.993, *p* < 0.001), March (−0.84 ± 0.27, Z = −3.082, *p* < 0.05) and April (−0.94 ± 0.26, Z = −3.609, *p* < 0.01). Positive social interactions were also lower in July than February (−0.86 ± 0.28, Z = −3.106, *p* < 0.05), and there was a trend towards them being lower in July than April (−0.73 ± 0.27, Z = −2.713, *p* = 0.07). Negative interactions were lower in March (−0.88 ± 0.24, Z = −3.715, *p* < 0.01) and April (−0.82 ± 0.22, Z = −3.650, *p* < 0.05) than February. There was also a trend towards lower negative interactions in June than February (−0.65 ± 0.24, Z = −2.727, *p* = 0.06).

#### 3.1.2. Interactions within Seal Dyads

Frequency of social interactions also differed significantly when looked at in terms of dyadic interactions. Dyad 6 (S3 and S4) engaged in more positive interactions per observation period than dyad 1 (S1 and S2; 1.49 ± 0.29, Z = 5.091, *p* < 0.001), dyad 2 (S1 and S3; 0.91 ± 0.27, Z = 3.354, *p* < 0.05), dyad 3 (S1 and S4; 1.32 ± 0.29, Z = 4.616, *p* < 0.001), dyad 4 (S2 and S3; 0.91 ± 0.27, Z = 3.354, *p* < 0.05) and dyad 5 (S2 and S4; 1.23 ± 0.28, Z = 4.362, *p* < 0.001). Dyad 6 also engaged in more negative interactions per observation period than dyad 1 (1.68 ± 0.48, Z = 3.510, *p* < 0.01) and dyad 5 (1.75 ± 0.48, Z = 3.621, *p* < 0.01).

**Figure 4:**
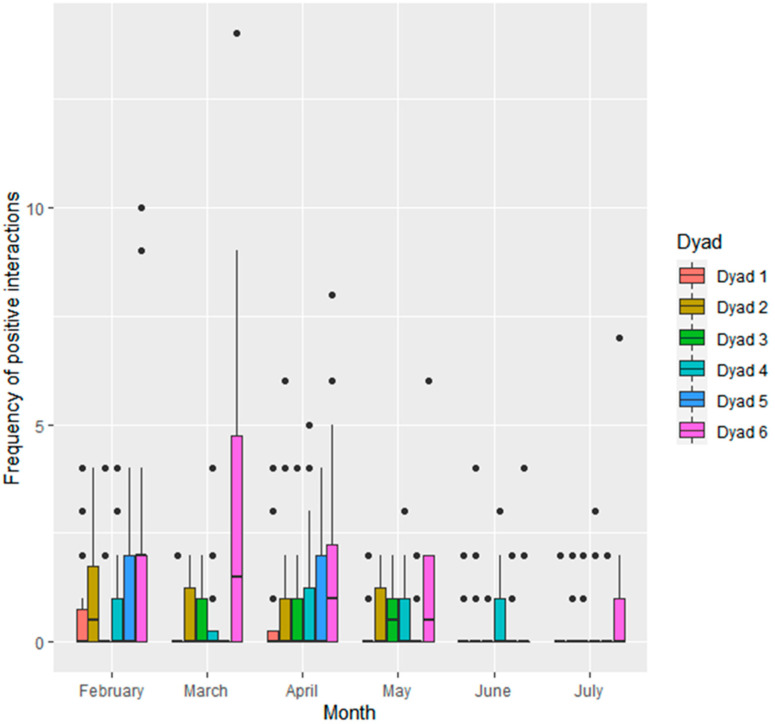
Frequency of positive social interactions given within each seal dyad (*n* = 6; dyad 1: S1 and S2, dyad 2: S1 and S3, dyad 3: S1 and S4, dyad 4: S2 and S3, dyad 5: S2 and S4, dyad 6: S3 and S4) per observation period.

**Figure 5:**
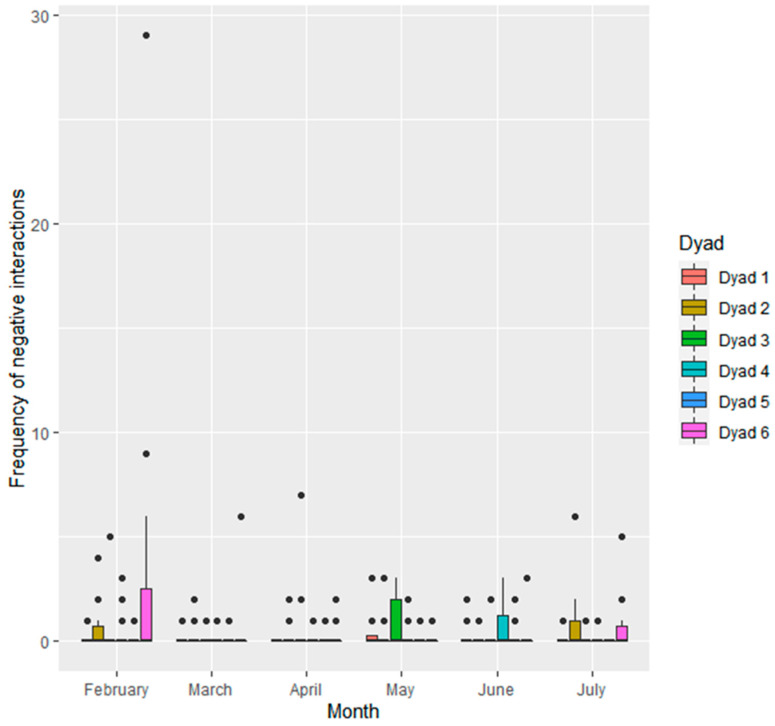
Frequency of negative social interactions given within each seal dyad (*n* = 6; dyad 1: S1 and S2, dyad 2: S1 and S3, dyad 3: S1 and S4, dyad 4: S2 and S3, dyad 5: S2 and S4, dyad 6: S3 and S4) per observation period.

Frequency of social interactions at the level of individual dyads also differed across the study months (Figure 4 and Figure 5). Positive interactions were lower in June than February (−0.70 ± 0.20, Z = −3.561, *p* < 0.01) and April (−0.61 ± 0.19, Z = -3.213, *p* < 0.05). There was a trend towards positive interactions being lower in June than March (−0.54 ± 0.20, Z = −2.686, *p* = 0.07) and lower in July than February (−0.56 ± 0.20, Z = 2.753, *p* = 0.06). Negative interactions were less frequent in March (−0.58 ± 0.2, Z = -2.832, *p* = 0.05) and April (−0.55 ± 0.19, Z = −2.833, *p* = 0.05) than February.

### 3.2. Fluidity in Group-Level Interactions over Time and Dyadic Reciprocity

Fluidity was observed in seal social interactions at a whole-group level. Positive social interaction networks were only consistent in March and May (r = 0.995, *p* < 0.05). Negative social interactions varied across all comparison points (*p* > 0.05). Dyadic reciprocity for positive social interactions was observed during February (r = 0.9376, *p* < 0.05), March (r = 0.9978, *p* < 0.05) and April (r = 0.9715, *p* < 0.05). Reciprocal relationships were not observed for positive social interactions from May to July (*p* > 0.05). Negative interactions were unbalanced across all study months (*p* > 0.05).

### 3.3. Nearest Neighbours

An overview of the average percentage of scans that seals were observed in close proximity to others within each observation period is detailed in Table 3. S1 was most frequently sighted alone (mean percent of time ± SD; 46 ± 24%), S2 was most frequently observed with S3 (34 ± 21% of time), S3 was most frequently observed with S4 (42 ± 22%) and S4 was most frequently observed with S3 (44 ± 23%).

Matrices based on nearest neighbours were fluid across all months, apart from February and March (r = 0.8611, *p* < 0.05) and March and May (r = 0.7427, *p* < 0.05), when nearest neighbour matrices were consistent. Nearest neighbours were reciprocal for all months (February: r = 0.9673, *p* < 0.05; March: r = 0.9858, *p* < 0.05; April: r = 0.8643, *p* < 0.05; June: r = 0.9887, *p* < 0.05; July: r = 0.9915, *p* < 0.05; August: r = 0.9674, *p* < 0.05), except for May (*p* > 0.05). With the exception of May, where nearest neighbour reflected positive interaction partners (r = 0.88, *p* < 0.05), no correlations were identified between nearest neighbours and positive social interaction partners (*p* > 0.05).

## 4. Discussion

Bachelor groups have been advocated as a zoological management tool which helps to support captive breeding programmes. Bachelor groups provide surplus males opportunities for socialisation [35], development of social skills [11] and in the case of younger males, to learn from older conspecifics [36,37], which is important for their welfare. Management of bachelor herds can be complicated by enclosure size/design limitations and behavioural incompatibilities between individuals [38]. Areas of importance in the successful formation and maintenance of bachelor groups include formation when animals are young in order to optimise group stability, limited inclusion of hand-reared individuals within the social group, exhibit designs which include opportunities for refuge, visual barriers and opportunities for separation of individuals where required [11]. Evidence-based management of social groups is extremely important in ensuring optimal welfare for zoo animals. This research contributes to our limited knowledge of SAFS social behaviour, and provides a baseline point for future work in this field, which has ramifications for the social management of this species.

### 4.1. Positive and Negative Social Interactions

Understanding social dynamics at a species-specific level helps to mitigate against management-related social disturbances in zoo-housed animals [17]. Social and play behaviours are indicative of positive affective states in zoo animals [39] and their absence may be indicative of problems in pinnipeds [9]. All individuals engaged in both positive and negative social interactions and were considered to be equal in terms of position within social networks. No extreme negative interactions were observed, and seals consistently engaged in more positive than negative interactions each month. Dyadic reciprocity was observed for frequency of positive social interactions across some but not all months. No dyadic reciprocity was seen in terms of frequency of negative interactions but nearest neighbours were largely reciprocal. In many bachelor groups, social hierarchies are used to mitigate social tension, with individuals engaging in social behaviour appropriate for their social rank [35]. Research into wild bachelor groups of New Zealand fur seals identified a tendency for non-physical interactions (e.g., vocalisation and ritualised dominance displays), which rarely escalated to aggression [40]. Relatedness and familiarity have been identified as potential drivers of success in bachelor groups of zoo-housed gorillas (*Gorilla gorilla gorilla*) [41]. No extreme aggression was seen and there was no evidence of a strong social hierarchy in this group. This may be due to the relatedness of the group and familiarity of members, or it may be that hierarchical behaviours were more subtle (e.g., dominance displays). Looking in greater depth at the types of negative behaviour and circumstances in which they occur in future work would enable a greater understanding of hierarchical structures in this and other groups of SAFSs, which would contribute to management which optimises animal welfare.

### 4.2. Temporality in Social Relationships

Longitudinal management of bachelor groups supports optimal species management [42], and the importance of understanding social networks of animals over time has been highlighted [18]. This research advocates such recommendations in SAFSs. Temporal changes in positive and negative social interactions were observed over the study months. However, the frequency of negative interactions did not increase in relation to the breeding season. Many polygynous species become highly aggressive and territorial during the breeding season [43]. However, in situ observations suggest that only low levels of interactions occur between non-breeding male SAFSs [8]. Within this bachelor group, individuals did not need to defend territories or fight over access to females, and this may explain the lack of relationship between time of year and frequency of negative interactions. Similar findings have been reported in zoo-housed western lowland gorilla bachelor groups [44].

The reasons for the temporal dynamics in relationships observed in this group are unclear. There may have been other confounding variables such as keeper routines, weather, or presence of the public which were beyond the scope of this research. Capturing data on temporal dynamics is important in understanding animal social networks [21]. Having a baseline understanding of the degree to which one expects seal social relationships to be flexible will enable early identification of deviations beyond the expected norm, which may be indicative of social complications within the group. This could occur as the seals age, or if there are any underlying illnesses or other problems. It is recommended that further research be undertaken in other facilities to determine the degree to which these results reflect other bachelor groups of SAFSs, especially when males are housed with non-kin. Furthermore, comparisons with social behaviour in female-only and mixed-sex groups will allow the development of evidence-based management protocols in SAFSs. Such knowledge could also be expanded into other pinniped species.

### 4.3. Factors Affecting Observed Social Interactions

In this study, individuals S3 and S4 engaged in more positive interactions than other dyads. S3 and S4 were the youngest members of the social group. S1, the oldest member of the social group, spent more time alone than S3 and S4. Age has been identified as an important driver of social relationships. Age could thus be driving the strong relationship and high frequency of interactions between S3 and S4. In gorillas, frequency of affiliative behaviour is reduced in individuals over 10 years old [11], and formation of positive long-term relationships is affected principally by familiarity and relatedness [41]. In wild golden snub-nosed monkeys (*Rhinopithecus roxellana*), similarly aged males that played together in breeding groups formed preferential relationships when they later joined a bachelor group [36]. In elephants, calves engage in most social interactions [45] and physical social interactions are reduced in older individuals [46], especially within bachelor groups [47].

There is also the potential for individual personalities to be impacting on these relationships. Individual personalities affect the experience of animals in zoo environments [48]. Personalities have been recognised in pinnipeds [49] and social preferences have been observed in breeding colonies of wild Galapagos sea lions (*Zalophus wollebaeki*) [1]. Personality has been identified as a driver of friendships [50], and it has been linked to sociability [51,52] and compatibility between individuals [53,54]. Age, rearing, relatedness, familiarity, relationship quality and enclosure design may all impact on the success of social groups and valence of social interactions in bachelor groups [11]. Across multi-sex social groups, age, familiarity and individual personalities have all been identified as having positive effects on individual compatibility [50,54,55,56]. The EAZA pinniped guidelines recommend consideration of the ‘character’ of male pinnipeds when designing social housing [9]. Future research should seek to understand factors which drive social relationships in SAFSs, particularly in bachelor groups, to enable the provision of social groups which optimise individual welfare.

### 4.4. Implications for Management of South American Fur Seals and Other Pinniped Species

Conflict avoidance techniques have been identified in a number of species, including rhesus macaques (*Macaca mulatta*), bonobos (*Pan paniscus*), chimpanzees (*Pan troglodytes*) [57] and gorillas [44]. In this study, the two seals who engaged in fewer social interactions also spent longer periods of time alone. There were no female SAFSs housed at Bristol Zoo at the time of this study, nor were there any other pinniped species on site. This means there was an absence of ‘high value’ resources (e.g., females). Research in gorillas indicates that males in bachelor groups engage in more displacement behaviour than males in breeding groups, and males in breeding groups engage in more non-escalated aggression than those in bachelor groups. These differences have been attributed to the lack of females in the bachelor group and employment of conflict reduction techniques in the breeding group [44]. Similar conflict reduction techniques may be being employed by this bachelor group of SAFSs. Adequate space to avoid others is advocated in bachelor group management [58] and it is highlighted in the EAZA pinniped management guidelines [9]. The opportunity to choose to interact with or avoid conspecifics is important for ensuring positive welfare in social animals [59]. The results of this work highlight the importance of providing environments which enable seals to execute conflict avoidance behaviours, thus ensuring successful SAFS bachelor group management.

Sociability can be identified via both physical interaction and proximity to others. Variability was seen between social interaction partners and nearest neighbours in this group of SAFSs. This variability highlights the need to fully understand social dynamics in SAFSs before making long-term management decisions. Proximity to group members and engagement in social interactions should both be considered in animal behaviour assessments. A sudden reduction in positive social interactions, increased time spent alone or increased negative social interactions, beyond the natural flexibility of the social group, may be a cause for concern [60]. Creating behavioural profiles which detail baseline social behaviour for individuals will allow early identification of potential problems in management of SAFSs and other pinniped species. Extreme individual behavioural changes, especially increases in negative interactions, not only impact the individual but it could affect the social stability of the entire group. Zoo researchers advocate understanding animal welfare at an individual level [59] and we support this recommendation in relation to SAFSs and wider pinniped social management.

Finally, it is important to recognise that this research was undertaken on a small sample of SAFSs at a single facility and thus may not be representative of wider SAFS management. However, the group itself was typical of the size of social groups SAFSs are reportedly housed in within ZIMS collections [20]. The study can thus be considered important in beginning to advance knowledge of social behaviour in this understudied species. To increase the applicability of these findings, it is advocated that future research is undertaken to understand how comparable the behaviour recorded here is to other bachelor groups and other types of socially housed SAFSs (e.g., mixed-sex and female-only groups) to determine the degree to which this social group is representative of other socially housed SAFSs, and how comparable SAFS social behaviour is to other more well-studied species (e.g., primates). If similar concepts are present and similar factors appear to be driving interactions within groups of SAFSs, this would allow wider application of the detailed primate literature. Expansion of this into pinniped species as a whole would be beneficial moving forwards to support in the development of evidence-based pinniped social management guidelines.

## 5. Conclusions

This research has highlighted the paucity of evidence-based knowledge on South American fur seal and wider pinniped species’ social behaviour within zoos. In the wild, SAFSs engage in complex social behaviour and spend large periods of time in extensive social groups in breeding rookeries. As a polygynous species, there may be a need to house surplus males in bachelor groups and understanding the implications of such management is important in ensuring positive welfare at an individual level. This research found that this group of SAFSs engaged in a range of positive social interactions and negative social interactions remained low throughout. Despite the consistent nature of this zoo social group (i.e., there were no changes in social group members during these observations), social interactions were not static. There were large amounts of individual variation and temporal dynamics were observed in social relationships. We have demonstrated the importance of longitudinal monitoring of social relationships, to establish baseline social behaviour profiles and support evidence-based species management. In particular, we recommend the monitoring of negative social interactions, as these may be indicative of problems developing within the social group. We advocate that this research is extended, to further develop our understanding of SAFS social needs within zoo environments, understand the differences between single-sex and mixed-sex groups and identify the degree to which the extensive research conducted in other polygynous species (e.g., gorillas) is applicable in the social management of these species moving forwards. Enhancing understanding of SAFS social needs will enable the development of evidence-based social management guidelines, which are imperative for ensuring optimal welfare for zoo-housed SAFSs.

## Figures and Tables

**Table 1:** South American fur seals at Bristol Zoo Gardens.

Seal ID	Sex	Age at Start of Study	Stage of Maturity	Relationship to Others
S1	M	11	Adult	Full brother to all
S2	M	9	Adult	Half-brother to all
S3	M	8	Adult	Full brother to all
S4	M	7	Adult	Full brother to all

**Table 2:** Ethogram detailing positive and negative behaviours recorded during the study. Ethogram was based on [25], and modified during preliminary observations prior to commencement of the study.

Interaction Category	Behaviour	Description
Positive	Nose–nose	Touching another’s face with their nose
	Nose–body	Touching another’s body (not the face/nose) with their nose
	Mouth–mouth	Gentle touching of another’s mouth with their mouth
	Play	Individuals jump, slide and porpoise round the enclosure. This may centre around enrichment
Negative	Bite	Individuals use teeth to touch another individual
	Display	Individuals have wide mouths and square each other up. May sway necks side to side opposite another individual
	Displacement	An individual approaches another causing them to move out of the way (by more than 1 body length). May result in approaching individual taking their place

**Table 3:** Average percentage of each observation period that seals were observed with others.

Focal Seal	Nearest Neighbour (Mean % ± SD)				Alone
	S1	S2	S3	S4	
S1		21 ± 17	21 ± 17	19 ± 15	46 ± 24
S2	20 ± 17		34 ± 21	23 ± 17	32 ± 17
S3	18 ± 17	30 ± 21		42 ± 22	21 ± 16
S4	18 ± 14	21 ± 15	44 ± 23		27 ± 17

## Data Availability

The data that support the findings of this study are available from the corresponding author upon reasonable request.
